# Estimation of myocardial deformation using correlation image velocimetry

**DOI:** 10.1186/s12880-017-0195-7

**Published:** 2017-04-05

**Authors:** Athira Jacob, Ganapathy Krishnamurthi, Manikandan Mathur

**Affiliations:** 1 Department of Engineering Design, Indian Institute of Technology Madras, Chennai, 600036 India; 2 Department of Aerospace Engineering, Indian Institute of Technology Madras, Chennai, 600036 India; 3 Department of Biomedical Engineering, The Johns Hopkins University, Baltimore, 21218 USA

**Keywords:** Tagged magnetic resonance imaging, Correlation image velocimetry, Cardiac deformation, Cardiac strain

## Abstract

**Background:**

Tagged Magnetic Resonance (tMR) imaging is a powerful technique for determining cardiovascular abnormalities. One of the reasons for tMR not being used in routine clinical practice is the lack of easy-to-use tools for image analysis and strain mapping. In this paper, we introduce a novel interdisciplinary method based on correlation image velocimetry (CIV) to estimate cardiac deformation and strain maps from tMR images.

**Methods:**

CIV, a cross-correlation based pattern matching algorithm, analyses a pair of images to obtain the displacement field at sub-pixel accuracy with any desired spatial resolution. This first time application of CIV to tMR image analysis is implemented using an existing open source Matlab-based software called *UVMAT*. The method, which requires two main input parameters namely correlation box size (*C*_*B*_) and search box size (*S*_*B*_), is first validated using a synthetic grid image with grid sizes representative of typical tMR images. Phantom and patient images obtained from a Medical Imaging grand challenge dataset (http://stacom.cardiacatlas.org/motion-tracking-challenge/) were then analysed to obtain cardiac displacement fields and strain maps. The results were then compared with estimates from Harmonic Phase analysis (HARP) technique.

**Results:**

For a known displacement field imposed on both the synthetic grid image and the phantom image, CIV is accurate for 3-pixel and larger displacements on a 512 × 512 image with (*C*_*B*_,*S*_*B*_)=(25,55) pixels. Further validation of our method is achieved by showing that our estimated landmark positions on patient images fall within the inter-observer variability in the ground truth. The effectiveness of our approach to analyse patient images is then established by calculating dense displacement fields throughout a cardiac cycle, and were found to be physiologically consistent. Circumferential strains were estimated at the apical, mid and basal slices of the heart, and were shown to compare favorably with those of HARP over the entire cardiac cycle, except in a few (∼4) of the segments in the 17-segment AHA model. The radial strains, however, are underestimated by our method in most segments when compared with HARP.

**Conclusions:**

In summary, we have demonstrated the capability of CIV to accurately and efficiently quantify cardiac deformation from tMR images. Furthermore, physiologically consistent displacement fields and circumferential strain curves in most regions of the heart indicate that our approach, upon automating some pre-processing steps and testing in clinical trials, can potentially be implemented in a clinical setting.

**Electronic supplementary material:**

The online version of this article (doi:10.1186/s12880-017-0195-7) contains supplementary material, which is available to authorized users.

## Introduction

Cardiovascular abnormalities are a major cause of disease in the present day. In addition to causing significant problems to the affected patients, treatment and further follow up after heart failure impose heavy expenses on the health care system. Identifying individuals at significant risk of developing heart failure is therefore of paramount importance.

Various imaging modalities can be used to assess global ventricular function through measures such as ejection fraction, end-systolic volumes etc. that give important information about the overall functioning of the heart [1]. These measures, though being strong predictors of cardiovascular disease, are often insensitive to regional myocardial dysfunction; for example, regional dysfunction can often be masked by a normal ejection fraction [2]. On the other hand, abnormalities in heart wall motion, often an indicator of ischemia caused by the occlusion of the coronary arteries, are a sensitive indicator and can be seen much before other symptoms set in [3, 4].

In general, heart wall motion abnormalities are considered to be life threatening. Several techniques, including tagged MRI, can provide robust quantification of such abnormalities and hence facilitate diagnosis. Thus regional performance markers such as quantification of strain and torsion have emerged as more accurate predictors of myocardial disease. This has further been validated by many studies that have found consistent differences in regional strains, strain rates and torsion between healthy and diseased myocardium [5, 6]. In addition to quantifying regional myocardial abnormalities, the inferred heart wall motion can also be used to model forces in the heart wall for realistic simulations of cardiac motion.

Currently, cardiovascular magnetic resonance (MR) is the gold standard for assessing global as well as regional heart function due to its high spatial and temporal resolution [7]. Various MR-based techniques have been proposed to infer cardiac deformation. The Displacement Encoding with Stimulated Echoes (DENSE) method relies on the modulation of phase according to position [8]. On the other hand, feature-tracking techniques [9, 10] track myocardial boundaries and other distinct landmarks to estimate displacements. Zerhouni et al. (1988) [11] introduced an MR based noninvasive method for imaging called tagged MRI (tMR). The tags, which are regions of reduced signal intensity in the images caused by the saturation of magnetic field in selective planes, act as intrinsic tissue markers. Since then, tMR techniques have shown great potential for measuring the local mechanical wall function and other cardiac abnormalities [12–19]. Despite these successful advances, tMR has not been adopted in everyday clinical use, partly because of the complicated post processing. Harmonic Phase analysis (HARP) [20] is currently the most widely used technique for quantifying myocardial motion from tMR images. The HARP method analyzes motion by filtering harmonic peaks in the frequency domain of the image and computes a dense displacement map of tag lines by tracking their phase changes over time.

In the well-established experimental technique called Particle Image Velocimetry (PIV) in experimental Fluid Mechanics, a fluid flow is typically seeded randomly with micron-size glass particles (in water) or oil droplets (in air). These particles are illuminated using a laser light sheet, and visualized in images acquired using a high-speed camera. Pulsed lasers are often used if the flow velocities are large or flow time scales are small. The patterns of the intensity distribution in the time series images are then tracked using correlation image velocimetry (CIV) to quanitatively estimate the velocity field in the flow [21]. Here, tracking tag lines and their intersections in tMR images is reduced to the problem of tracking patterns in the intensity distribution over successive images, performed using CIV. More details on CIV are given in the CIV Implementation subsection of the “Methods” section, and the Additional file 1: Appendix. We adapt an existing Matlab-based PIV tool called *UVMAT* [22] to calculate a dense myocardial displacement field in cardiac tMR images.

In this paper, we aim to adapt correlation image velocimetry to estimate displacements and strain fields from tagged-MRI images. Furthermore, we verify that the estimated displacement and strain fields compare favorably with ground truth and HARP, respectively using a publicly available database of 15 healthy individuals. Our specific objectives in this paper are:

- To demonstrate the first-time use of CIV with extracted tag lines as input to quantitatively estimate cardiac deformation fields from tMR images,
- To characterize the errors in displacement and strain estimation using synthetic and phantom grids and identify the optimum CIV algorithm parameters to minimize error,
- To validate the CIV-estimated dense displacement and strain fields via comparisons with ground truth and HARP, respectively.

## Methods

### Patient data

**Fig. 1:**
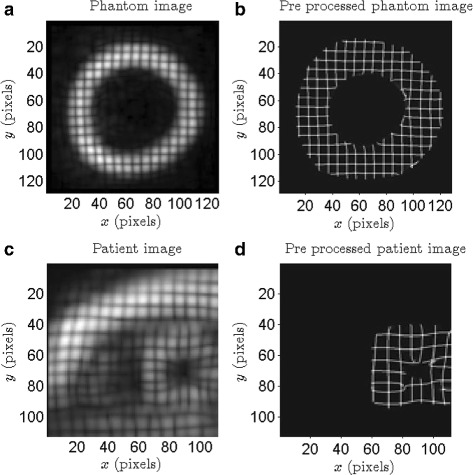
**a** 128 × 128 phantom image, **b** pre-processed phantom image, **c** 112 × 112 patient image, **d** pre-processed patient image. The images shown in 1a & b contain square grids of size around 6 pixels, and the corresponding bounding lines are approximately 2 pixels thick

The tMR images of the phantom (Fig. 1 a) and patients (Fig.1 c) used in this study are from the “Statistical Atlases and Computational Models of the Heart: Imaging and Modelling Challenges” (STACOM) challenge held during the 2011 edition of the Medical Image Computing and Computer Assisted Intervention (MICCAI) Conference [23]. The 4D tMR sequence was obtained with three sequential breath hold acquisitions in each orthogonal direction (TR/TE = 7.0/3.2 *ms*, flip angle =19−25°, tag distance: 7 *mm*)[24]. Images were acquired with reduced field-of-view enclosing the left ventricle (LV; 108x108x108 *mm* ^3^); the voxel dimension of each image is 112 × 112 × 111 pixels. The images are also supplemented by positions of landmark points tracked by two independent observers over an entire cardiac cycle, henceforth referred to as ground truth. *The data is hosted by the Cardiac Atlas Project. It was acquired at the Division of Imaging Sciences and Biomedical Engineering, King’s College London, United Kingdom, and the Department of Internal Medicine II - Cardiology, University of Ulm, Germany, with ethics committee and patient approval. More details on the data can be found in* [23].

### Grid extraction

The tMR short-axis slices (112 × 112 pixels) are pre-processed to extract the tag grid. The tag grid extraction is based on the HARP technique [20]. A windowing function *w*(*i,j*)=(1− sin(*π*(*r*−6)/2))/2 centered at the lowest harmonic frequency in the Fourier transform image was used to isolate the spectral peak; here, *r* is the distance from the center. The inverse Fourier transform returns a complex harmonic image from which the harmonic phase image is estimated using the inverse tangent function. The line discontinuities in the harmonic phase image correspond to the location of the tag lines, and are localized using a standard Laplacian edge detection technique. This process was repeated for the two tag directions. The edge images are then multiplied and intensity adjusted using the Matlab function imadjust() (with default parameters) to obtain the tag grid images shown in Fig. 1 b & d. The tag grid images are then interpolated onto a grid of size 512 × 512 from the original sizes of 128 × 128 and 111 × 112 (in pixels) for phantom and patient images, respectively. In the resulting image, the gray scale background is eliminated and the deformed tag grid is retained. The absence of a continuously varying background makes the images amenable to methods that rely on cross-correlation to track deformations at the sub-pixel level.

#### CIV implementation

The CIV algorithms described in [25] are implemented in the Matlab-based PIV software *UVMAT*, which also provides related tools like scanning of images and data files, geometric calibration, image pre-processing and analysis of time series. The CIV algorithm uses direct cross-correlation between a pair of images to perform pattern matching. Implemented as a hierarchical process, the iterative algorithm takes into account local pattern deformation by both strain and rotation. The image is divided into sub-regions, referred to as the correlation box in the rest of the paper, and a single displacement vector is computed for every correlation box. The correlation box size *C*_*B*_ typically has to be large enough to contain sufficient number of features and small enough for the displacement field not to vary too strongly within a correlation box. The displacement vector for every correlation box is obtained by determining the peak location with sub-pixel accuracy in the cross-correlation plotted as a function of the relative shift between sub-regions in a pair of images. It is noteworthy that the algorithm accounts for the leading order variation of the displacement field within a correlation box. The range of relative shifts that are considered is referred to as the search box size *S*_*B*_. The spatial resolution of the final displacement field is determined by *C*_*B*_ and the overlap between neighbouring correlation boxes, which is chosen to be 50% in all our calculations. The optimum values of *C*_*B*_ and *S*_*B*_ depend on the specific application, and we identify the optimum values for our tMR analysis in the first subsection of the “Results” section. The various steps involved in the CIV-processing of a given pair of images and the details of the CIV algorithm are described in the Additional file 1: Appendix. Finally, the temporal resolution of the estimated displacement fields can be as small as the temporal resolution of the image acquisition. In other words, a displacement field and a corresponding strain map can be computed for every image pair in the set of acquired images. For instance, in the patient data we analyse in this paper, if 20 images are available within a cardiac cycle, then one can obtain 20 different spatially resolved displacement fields within a cardiac cycle.

#### Post processing of displacement field

Myocardial strain maps for each frame are estimated from the estimated displacement fields. The displacement fields are first transformed from Cartesian coordinates to a cylindrical polar coordinate frame that is related to the anatomy of the LV. If *u, v* are displacements in Cartesian coordinates, the displacements in the cylindrical (*r*,*θ*) coordinate frame are calculated as

1 $$\begin{array}{@{}rcl@{}} u_{r} = u\cos\theta + v\sin\theta, \end{array} $$

2 $$\begin{array}{@{}rcl@{}} u_{\theta} = -u\sin\theta + v\cos\theta, \end{array} $$

where *u*_*r*_ and *u*_*θ*_ represent radial and circumferential displacements. The radial and circumferential strains in the cylindrical coordinate frame are, respectively, calculated as

3 $$\begin{array}{@{}rcl@{}} \epsilon_{rr} &=& \frac{\partial u_{r}}{\partial r},  \end{array} $$

4 $$\begin{array}{@{}rcl@{}} \epsilon_{\theta\theta} &=& \frac{1}{r}\frac{\partial u_{\theta}}{\partial \theta}+\frac{u_{r}}{r}.  \end{array} $$

Strain curves for the entire cardiac cycle are obtained using a cumulative estimate of the strains (Eqs. 3 & 4), starting at end diastole. Furthermore, we also calculate the divergence field *E*=*∂* *u*/*∂* *x*+*∂* *v*/*∂* *y* to quantify the compressibility of the myocardial tissue [26].

**Fig. 2:**
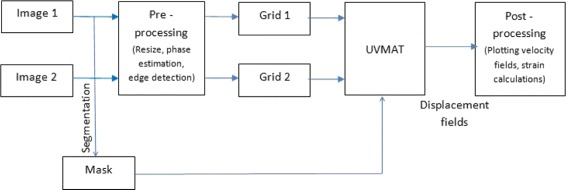
Workflow Pipeline

In summary, every image pair, comprising Image 1 and Image 2 separated by a time interval *Δ* *t*, is taken through the pipeline shown in Fig. 2 to obtain a displacement field that occurs in Image 1 to transform to Image 2.

### Numerical experiments

**Fig. 3:**
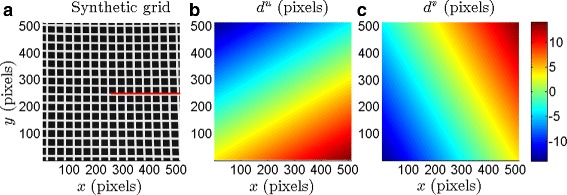
**a** 512 × 512 synthetic grid image with grids of dimensions 23 ×23 pixels, bounded by 8 pixels thick white lines. The red horizontal line represents the cross-sectional cut used to estimate the errors shown in Fig. 4 a and b. Imposed displacement field: **b** *d* ^*u*^(*x,y*), the *x* component and **c** *d* ^*v*^(*x,y*), the *y* component, as described by Eqs. (5) and (6). The minimum and maximum values of |*d*_*u*_| are 0 & 11 pixels, respectively; the corresponding values for |*d*_*v*_| are 0 & 9 pixels

Typically, PIV involves analysis of images of particles seeded in a fluid flow, with around 5 to 10 particles occupying every correlation box. To validate the use of CIV algorithms to quantify motion in grid-like images, we perform a systematic quantitative analysis of the errors associated with our estimates of known displacement fields. The error analysis is done for two kinds of images: (i) a noise-free 512 × 512 image of a black and white regular grid (Fig. 3 a) that is similar to the grids formed by the tag lines in the 512 × 512 interpolated patient images, and (ii) the pre-processed phantom image shown in Fig. 1 b. The images are subject to the following displacement field that represents a simplified model of the heart motion:

5 $$\begin{array}{@{}rcl@{}} u_{r} &=& 0.02r,  \end{array} $$

6 $$\begin{array}{@{}rcl@{}} u_{\theta} &=& 0.035r, \end{array} $$

where $r = \sqrt {(x-256)^{2} + (y-256)^{2}}$ and *θ* represent the radial and azimuthal coordinates of a cylindrical polar coordinate system with its origin at (256,256) pixels. The radial (*u*_*r*_) and rotational (*u*_*θ*_) components represent the relaxation and torsion of the heart during the cardiac cycle. The expression for *u*_*θ*_ in Eq. (6) represents an overall rotation of 2° in a time interval of *Δ* *t*=1 unit.

The displacement field described by Eqs. (5) and (6) are then imposed on the chosen original image (Image 1) to obtain the derived image (Image 2). The image pair is processed using different values for the correlation box size (*C*_*B*_) and the search box size (*S*_*B*_) to identify an optimal (*C*_*B*_,*S*_*B*_) combination that minimizes the error in the estimated displacement fields. The resulting optimal values of (*C*_*B*_,*S*_*B*_) are then used to process the patient images.

## Results

### Synthetic grid with analytical displacement field

We start by quantifying the errors in the estimated displacement fields on the synthetic image shown in Fig. 3 a. The synthetic displacement field described by Eqs. (5) & (6) is imposed on the synthetic image. The distribution of the resulting horizontal and vertical pixel displacements (*d*_*u*_ and *d*_*v*_) are shown in Fig. 3 b and 3 c, respectively. Specifically, the intensity distribution *I*_*o*_(*x,y*) in Fig. 3 a is related to the intensity distribution *I*_*d*_(*x,y*) of the derived image by *I*_*d*_(*x*+*d*_*u*_,*y*+*d*_*v*_)=*I*_*o*_(*x,y*). The original image in Fig. 3 a and the derived image are then used as Image 1 and Image 2, respectively for the CIV analysis.

The CIV analysis was run using different values of *C*_*B*_ (40≤*C*_*B*_≤85) and *S*_*B*_ (15≤*S*_*B*_≤40). For each pair of values (*C*_*B*_,*S*_*B*_), we calculate the horizontal and vertical displacement fields denoted by $d_{c}^{u}$ and $d_{c}^{v}$, respectively. The error in $(d_{c}^{u},d_{c}^{v})$ is then quantified as:

7 $$ e^{u}_{s} = \frac{|s^{u}-s_{c}^{u}|}{s^{u}},   $$

where $s_{c}^{u}$ is the slope of the best-fit straight line corresponding to the variation of $d_{c}^{u}$ with *x*, along the red horizontal line shown in Fig. 3 a. The error $e^{v}_{s}$ associated with the slope in $d_{c}^{v}$ is defined in a similar manner. For the imposed displacement fields, the exact values of the slopes are *s* ^*u*^=0.02 and *s* ^*v*^=0.035. For a given pair of values for (*C*_*B*_,*S*_*B*_), we also define the horizontal and vertical displacement errors at each pixel location (*x,y*):

8 $$ e^{u} = \left|\left(d_{u}-d^{u}_{c}\right)/d_{u}\right|,   $$

with the corresponding vertical displacement error being $e^{v} = |(d_{v}-d^{v}_{c})/d_{v}|$.

**Fig. 4:**
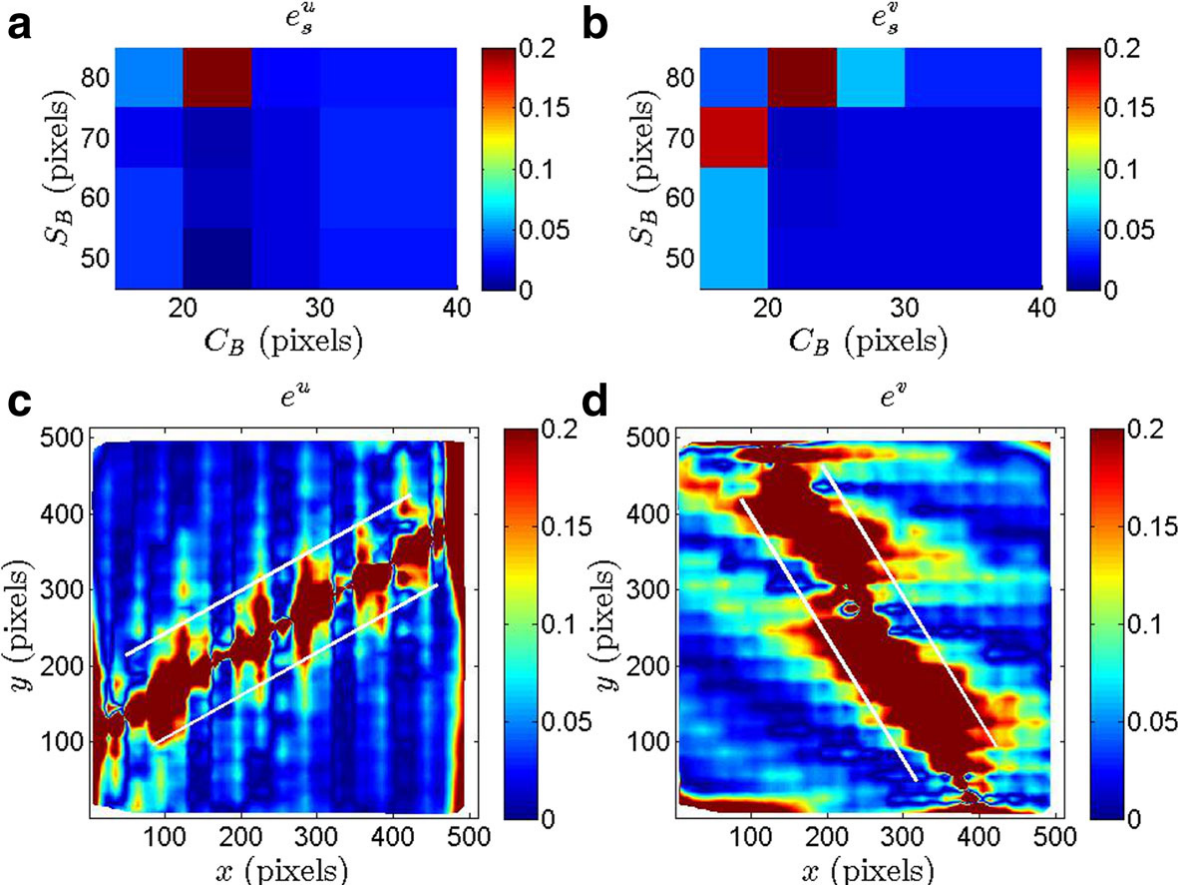
Errors corresponding to the estimated displacement fields obtained from a CIV analysis of the synthetic grid images with an imposed analytical velocity field. **a** Error in slope ($e^{u}_{s}$, defined in Eq. 7) plotted as a function of *C*_*B*_ and *S*_*B*_. **b** $e^{v}_{s}$ plotted as a function of *C*_*B*_ and *S*_*B*_. **c** Error in the horizontal displacement field (*e* ^*u*^, defined in Eq. 8) plotted at every pixel location (*x,y*) for (*C*_*B*_,*S*_*B*_)=(25,55). **d** Error in the vertical displacement field (*e* ^*v*^) plotted at every pixel location (*x,y*) for (*C*_*B*_,*S*_*B*_)=(25,55). The white lines in (**c**) and (**d**) correspond to |*d* ^*u*^|=3 pixels and |*d* ^*v*^|=3 pixels, respectively. The colorbars in (**c**) and (**d**) are saturated at an error of 0.2 to bring out the features in the plots clearly

Figure 4 a shows the distribution of $e^{u}_{s}$ as a function of *C*_*B*_ and *S*_*B*_; the corresponding variation of $e^{v}_{s}$ as a function of *C*_*B*_ and *S*_*B*_ is shown in Fig. 4 b. The errors $e^{u}_{s}$ and $e^{v}_{s}$ are relatively small over a reasonably wide range of *C*_*B*_ and *S*_*B*_; for small *C*_*B*_ and large *S*_*B*_, however, the errors are as large as 20%. On the (*C*_*B*_,*S*_*B*_) plane, *C*_*B*_=25 pixels and *S*_*B*_=55 pixels corresponds to the least errors, with $e^{u}_{s} = 1.7$% and $e^{v}_{s} = 1.7$%. We verified that (*C*_*B*_,*S*_*B*_)=(25,55) pixels corresponds to small errors (∼ 5%) in estimating the slope of the displacement variations along a few other lines (that are not necessarily horizontal) as well. We now proceed to present the errors associated with (*C*_*B*_,*S*_*B*_)=(25,55) pixels in more detail.

Figure 4 c and d show the distribution of *e* ^*u*^ and *e* ^*v*^, respectively, as a function of pixel location (*x,y*) for (*C*_*B*_,*S*_*B*_)=(25,55) pixels. We recall that the imposed horizontal and vertical displacement fields are shown in Fig. 3 b and c, respectively. The horizontal displacement error is small (≲5%) in large parts of the domain except along a thin patch that is roughly aligned along the axis given by *y*=0.57*x*. Upon further investigation, we find that *e* ^*u*^ is large in regions where |*d* ^*u*^| is small as confirmed by the contour lines bounding the region corresponding to |*d* ^*u*^|≤3 pixels in Fig. 4 c. Indeed, there is an evident correlation between small magnitudes of the imposed displacements and relatively large error in the estimated displacements.

The distribution of *e* ^*v*^ shown in Fig. 4 d is qualitatively consistent with that of *e* ^*u*^, with *e* ^*v*^ being relatively large in regions of small imposed vertical displacement. The contour lines bounding the region |*d* ^*v*^|≤3 pixels (obtained from the distribution of *d* ^*v*^ shown in Fig. 3 c) are shown in Fig. 4 d, clearly demarcating the domains of large and small vertical displacement error. It is noteworthy that the region of small |*d* ^*v*^| is aligned with *y*=−1.75*x*, which is the line along which *d* ^*v*^ is zero. In summary, based on Fig. 4, we conclude that displacements that are 3 pixels or more in 512 × 512 grid-like images are accurately determined when the CIV-processing parameters correspond to (*C*_*B*_,*S*_*B*_)=(25,55) pixels.

### Phantom images with analytical velocity field

Using the optimal values of (*C*_*B*_,*S*_*B*_)=(25,55) pixels identified in the previous subsection, we perform the error analysis on phantom images. We start with the raw phantom image shown in Fig. 1 a, interpolate it on to a 512 × 512 pixels image, and then impose the displacement field described by Eqs. 5–6. The original and derived images are than pre-processed to obtain two 512 × 512 pixels pre-processed phantom images.

**Fig. 5:**
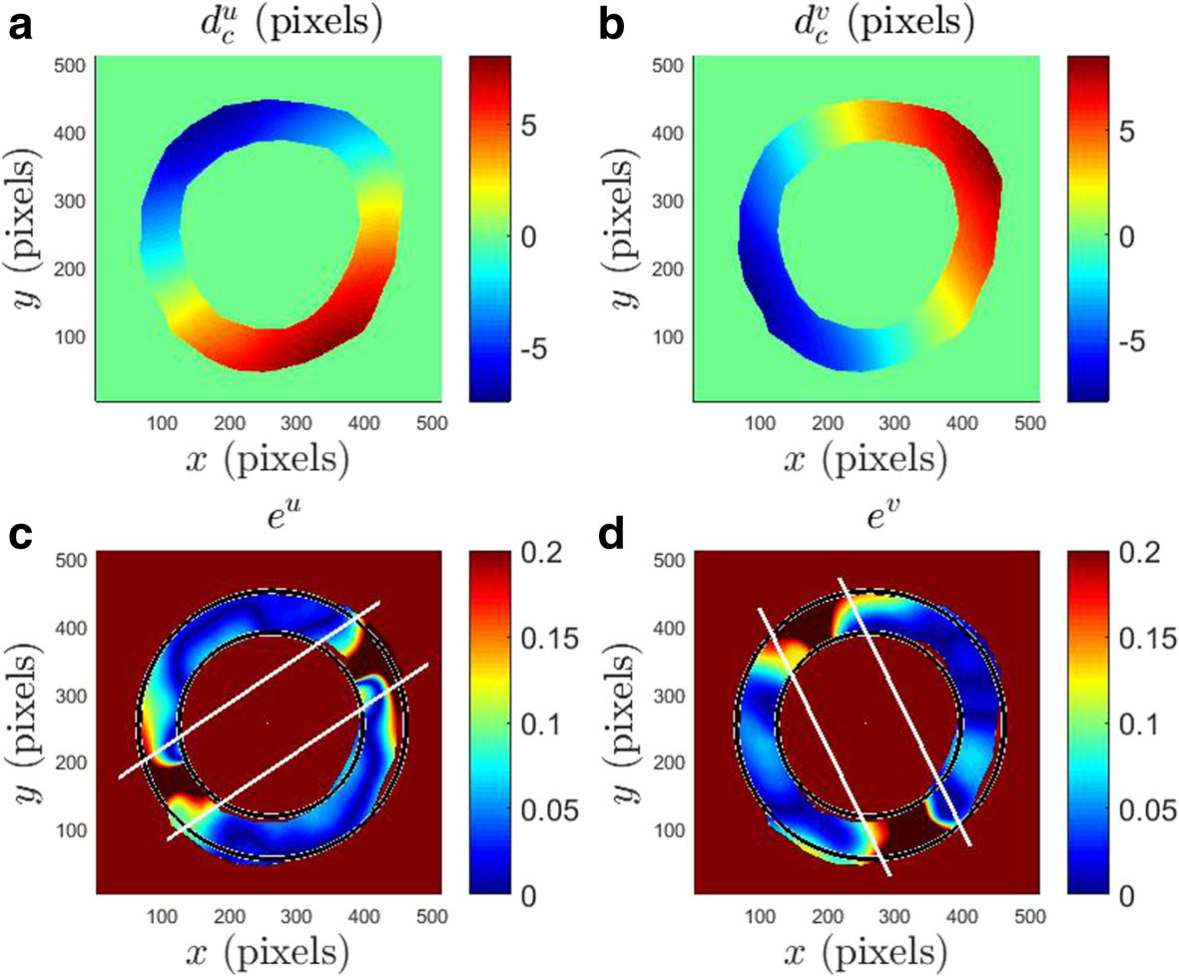
The estimated displacement fields using (*C*_*B*_,*S*_*B*_)=(25,55) pixels: **a** $d_{c}^{u}$, **b** $d_{c}^{v}$ as a function of *x* and *y* on the 512 × 512 pixels phantom image. The imposed displacement fields are shown in 3 b–c. **c** Error *e* ^*u*^ in the horizontal displacement field plotted at every pixel location (*x,y*) for (*C*_*B*_,*S*_*B*_)=(25,55). **d** Error in the vertical displacement field (*e* ^*v*^) plotted at every pixel location (*x,y*) for (*C*_*B*_,*S*_*B*_)=(25,55). The *white lines* in **c**) and **d**) correspond to |*d* ^*u*^|=3 pixels and |*d* ^*v*^|=3 pixels, respectively. The boundaries of the myocardium, i.e. the grid-like region in the 512 × 512 pixels phantom image are marked approximately by the black circles. The colorbars in **c**) and **d**) are saturated at an error of 0.2 to bring out the features in the plots clearly

Figure 5 a and b show the estimated horizontal ($d_{c}^{u}$) and vertical ($d_{c}^{v}$) displacement fields, respectively. The qualitative patterns of distribution of the imposed displacement fields, *d* ^*u*^ and *d* ^*v*^ shown in Fig. 3 b) and c), are captured well by the computed displacement fields, $d^{u}_{c}$ and $d^{v}_{c}$. We recall that the pre-processed phantom image (Fig. 1 b) contains grid lines in only an annular-like domain, and therefore it is important not to associate much significance to the estimated displacements in the non-grid, i.e. dark regions in the images.

In Fig. 5 c–d, we plot the relative errors *e* ^*u*^ and *e* ^*v*^ as a function of the pixel location *x* and *y*. The grid-like region, i.e. the boundaries of the myocardium are indicated by the black curves in Fig. 5 c–d. Both *e* ^*u*^ and *e* ^*v*^ are of relatively small magnitude for most of the domain inside the myocardium except for the region bounded by the white lines, which encompass the regions of |*d* ^*u*^|≤3 pixels and *d* ^*v*^≤3 pixels in Fig. 5 c & d, respectively. In summary, our analysis on the phantom images strengthened the main conclusion from the synthetic grid analysis that estimated displacements larger than 3 pixels in 512 × 512 grid-like images are accurate for (*C*_*B*_,*S*_*B*_)=(25,55) pixels.

The performance of CIV in terms of errors in estimated displacement for synthetic and phantom grids is summarised in Table 1. Specifically, the average error in horizontal and vertical displacements is less than 4 and 6%, respectively, if the corresponding displacements are larger than 3 pixels.

**Table 1:** A summary of relative errors in the CIV-estimated displacement fields for synthetic and phantom grids in regions of pixel displacement being 3 or more

	mean (*e* ^*u*^) (%)	std (*e* ^*u*^) (%)	mean (*e* ^*v*^) (%)	std (*e* ^*v*^) (%)
Synthetic grid	2.9	2.3	5.7	3.9
Phantom grid	3.5	2.9	3.6	3.2

### Patient data

#### Displacement field

**Fig. 6:**
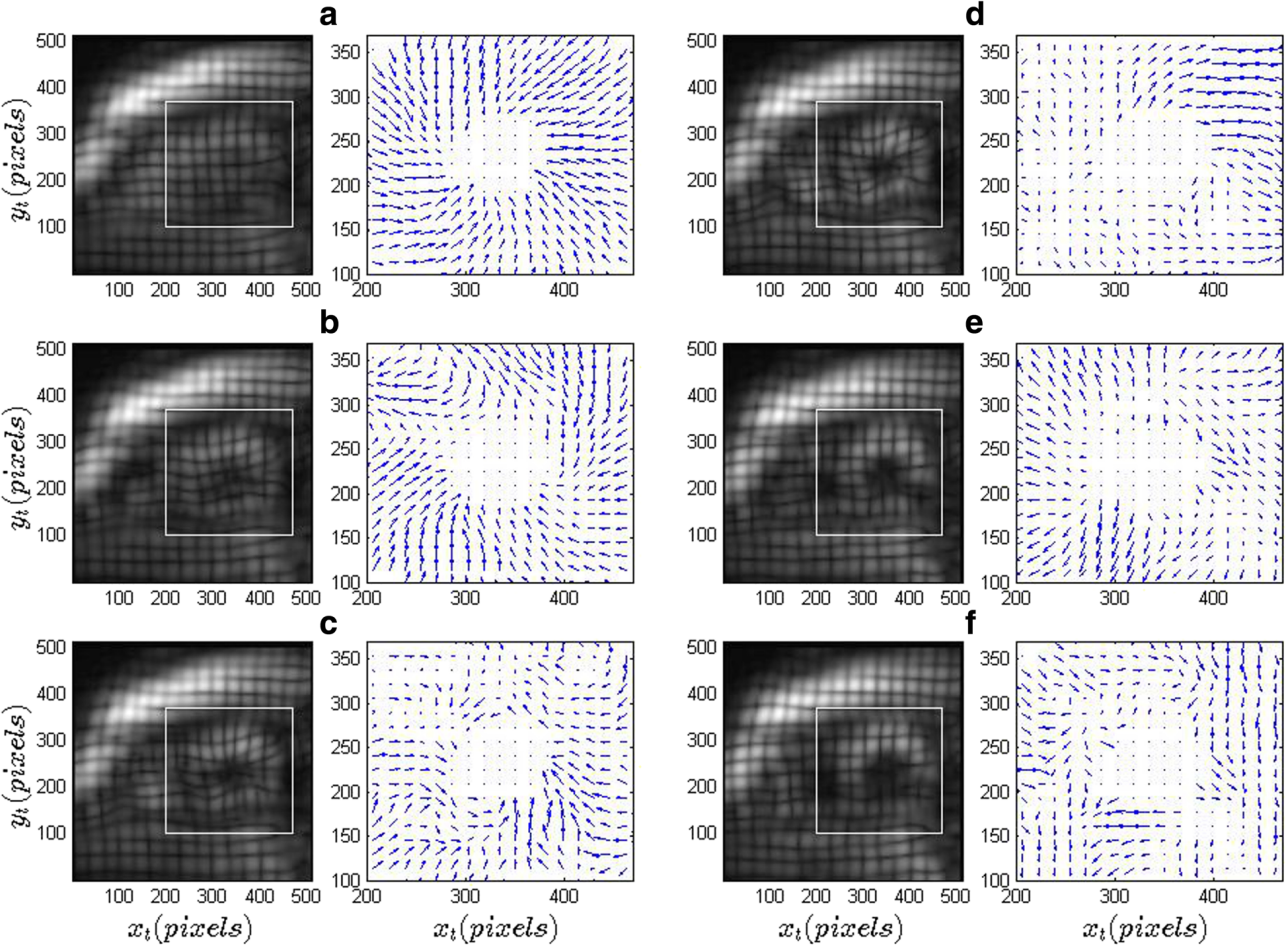
Dense displacement fields through a full cardiac cycle in patient-1 of the Challenge dataset. The images in (**a**)–(**f**) correspond to six different phases of the cardiac cycle, with (**a**)–(**c**) being systole. The *white boxes* in each of the images bound the myocardium, and only the displacement field within these boxes are shown. The *arrows* indicate the direction of the net displacement at the corresponding pixel location, and the *arrow* length is proportional to the magnitude of displacement

Figure 6 shows dense displacement fields of the myocardial tissue as calculated from an apical slice using (*C*_*B*_,*S*_*B*_)=(25,55) pixels. Each of the displacement fields in Fig. 6 has been obtained using pairs of images such that the average pixel displacement is 3 or more, as identified for error minimization in the previous two subsections. Specifically, we considered two points in the cardiac cycle separated by approximately 130ms for an image pair. Figure 6 a–c, representing the systolic phase of the cardiac cycle, show a mixture of anti-clockwise rotation (as experienced by an apical slice when viewed from the apex) and radially inwards motion as can be expected from a contracting heart. Figure 6 d–f represent diastole and the displacements show corresponding clockwise, radially outwards motion.

#### Landmark tracking

**Fig. 7:**
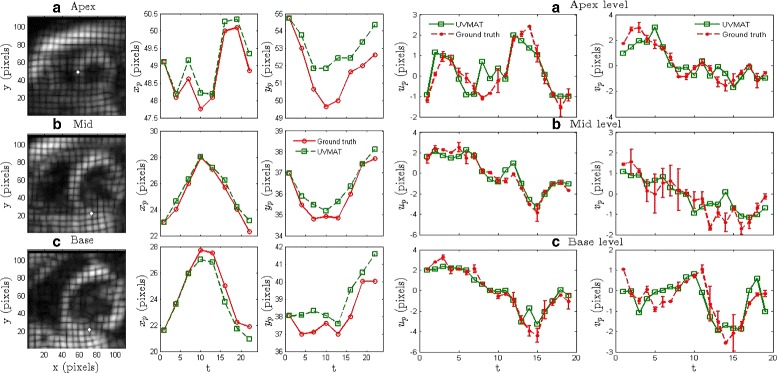
Tracking landmark positions (shown as white dots in the first column of images) at each heart level (**a**) Apex **b**) Mid **c**) Base over a full cardiac cycle. The *red* and *green lines* represent ground truth and calculated positions, respectively. The *x* and *y* coordinates of the landmark position, indicated using the subscript *p*, are plotted in the second and third columns, respectively. Comparing ground truth (*red*) and calculated (*green*) landmark instantaneous displacements at each heart level over a full cardiac cycle. Ground truth includes data from two separate observers to show inter-observer variability. Both *x* (*fourth column*) and *y* (*fifth column*) components are plotted, with the error bars being based on inter-observer variability. The time *t*=0 corresponds to end diastole in all the plots

For further validation, we tracked the positions of landmarks given along with the data set and compared our results with the ground truth. Landmark positions at any phase of the cardiac cycle are updated by adding the estimated instantaneous displacement to the current location. Instantaneous displacement at the landmark locations are obtained using spatial interpolation of the estimated displacement fields at the corresponding time. As shown in 2 ^*nd*^ and 3 ^*rd*^ columns of Fig. 7, our results are close to the ground truth without any significant bias or accumulation of errors over the cardiac cycle. Specifically, the base and the mid level landmark locations are tracked better than at the apex. The errors at the apex are possibly due to three-dimensional effects.

In addition to the positions of the landmarks, we also compared the estimated instantaneous displacements (at the ground truth landmark locations) to the ground truth landmark displacements (4 ^*th*^ & 5 ^*th*^ columns of Fig. 7). The estimated displacements largely fall within the inter-observer variability in the ground truth.

#### Strain rate estimation

**Fig. 8:**
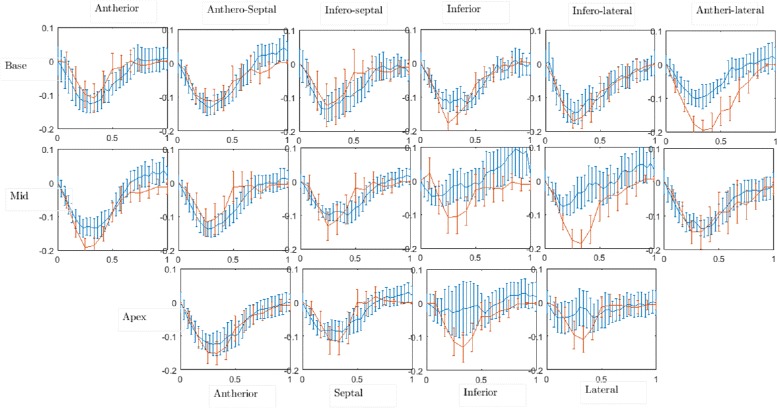
Comparison between CIV (*shown in red*) and HARP (*shown in blue*) for estimated mean (across 15 healthy individuals) circumferential strain in various segments of the heart. The *x*-axis of every plot signifies one cardiac cycle, with *x*=0 corresponding to end diastole. The error bars are based on the standard deviation across the 15 individuals

**Fig. 9:**
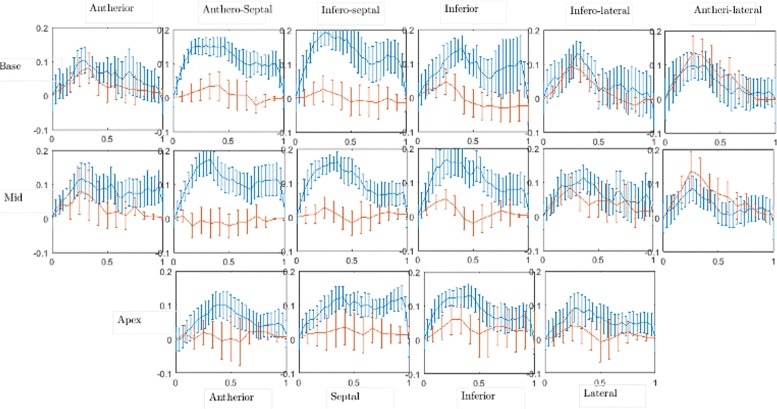
Comparison between CIV (*shown in red*) and HARP (*shown in blue*) for estimated mean (across 15 healthy individuals) radial strain in various segments of the heart. The *x*-axis of every plot signifies one cardiac cycle, with *x*=0 corresponding to end diastole. The error bars are based on the standard deviation across the 15 individuals

Radial and circumferential strain curves, as described in the “Methods” section, were derived from the calculated dense displacement fields for every patient. To obtain the strain curves at the basal, mid and apical slices, the myocardium is divided into segments according to the American Heart Association (AHA) 17-segment model [27]. Strain values are averaged over each anatomical segment, and then plotted as a function of time in Figs. 8 and 9. A similar analysis was performed using an evaluation version of the commercial implementation of the HARP algorithm, and the results are compared with those of CIV in Figs. 8 and 9. Our estimated circumferential strain curves (red) in Fig. 8 are in excellent quantitative agreement with HARP (blue); the standard deviation, calculated over all the patients, from the two methods are also of a similar magnitude. The mean absolute deviation of our circumferential strain curves from those of HARP across all times, segments and patients is 0.034, with the corresponding standard deviation being 0.024. The circumferential strain curves are predominantly negative, characteristic of contraction of the myocardium during systole. Radial strain curves (Fig. 9) are also observed to be physiologically consistent in the antherior, infero-lateral and antheri-lateral segments, i.e. increasing positive values during the first half of the cardiac cycle and then a subsequent decrease in the second half. CIV, however, underestimates the magnitudes of the radial strain in certain segments, which we discuss further in the next section. For the radial strain, the mean and the standard deviation of the absolute difference between CIV and HARP are 0.0625 and 0.038, respectively.

The compressibility associated with the estimated displacement fields can be used to validate their physical relevance in representing myocardial motion. The myocardium could be considered incompressible, and analysis of our results indicate that the volume change obtained from our estimated displacement fields are around 5% during systole, thus reasonably satisfying the incompressibility constraint. The estimates are obtained for 2D sections and is expected to vary if the displacement fields are estimated on 3D volumes.

## Discussion

One of the reasons for tMR not being used in routine clinical practice is the lack of easy-to-use tools for image analysis and strain mapping. In this paper, we have presented the performance analysis of a CIV algorithm, used often in experimental fluid mechanics, applied to tMR image analysis. We first characterized the accuracy of the CIV algorithm using simulated displacement fields, and then tested it on the STACOM 2011 data set. The algorithm was able to recover the simulated displacement fields superimposed on a synthetic 2D grid as well as on phantom and patient images. The errors in the estimation of the displacement field were found to be within the limits of error reported widely in literature.

The CIV algorithm requires the values of *C*_*B*_ (the correlation box size) and *S*_*B*_ (the search box size) as inputs for estimating the displacement field. Using simulated displacement fields imposed on synthetic and phantom images, we determined the error in the estimated displacement fields as a function of *C*_*B*_ and *S*_*B*_. Our approach was shown to accurately estimate displacements of 3 pixels or more in 512 × 512 grid-like images, thus requiring us to choose time points in the cardiac cycle that are within the sensitivity limits of the algorithm. Depending on the imaging sequence, tag, grid and overall image sizes, a calibration study as described in this paper would be required to determine the optimum values of *C*_*B*_ and *S*_*B*_ that correspond to least errors. In addition to validating the method on simulated displacement fields, we tracked individual points in the myocardium across the cardiac cycle using CIV to find near-perfect agreement with the expert tracked ground truth.

Following validation and appropriate input parameter selection, we estimated displacement fields from patient data. The estimated displacement fields capture the periodic physiologic motion of the heart accurately. The smoothness of the estimated displacement fields indicate the effectiveness of the outlier rejection techniques and smoothing techniques implemented in *UVMAT*. The displacement vectors were estimated on 100x100 points on the overall grid and later interpolated for pixel-wise fields. It is also possible to increase the density of points on which the displacement fields are estimated, but at the cost of increased computational time.

The estimated Eulerian strain trends are consistent with physiology, i.e. predominantly negative (positive) circumferential (radial) strain in the entire cardiac cycle starting at end diastole, thus making our approach potentially useful for diagnosis. The strain curves are determined in sixteen segments of the AHA 17-segment model of the heart. Despite the noise arising due to finite difference approximation in the Eulerian strain estimate, post-processing of the estimated displacement field allows us to determine strain curves from different segments of the heart.

An important pre-processing step is myocardial segmentation. For high throughput it is essential to have an algorithm that can provide a 3D segmentation mask for all volumes in the cardiac cycle. A novel technique exploiting dynamic programming and image based cues has been developed recently [28]. However, in this work, a manual over-segmented mask is used to delineate the myocardium excluding the LV blood pool. An oversegmenting mask is defined so that it can be used on all cross sections of the heart and across the entire cardiac cycle.

The segmentation step can have an impact on the estimated displacement fields especially at the edges of the epicardium and endocardium because of the presence of discontinuities. Since the mask we used could include non-myocardial regions in some of the frames, and hence track unphysical deformation of the tag lines, it would be prudent to manually define (preferably by an expert) an accurate mask for every frame. We note, however, that accurate circumferential strain estimates in most regions of the heart indicate that an oversegmented mask does not have a serious impact in regions of large strain magnitudes. Radial strain estimates, however, are impacted by the boundary effects.

The results in this paper suggest that CIV is a viable tool for cardiac deformation analysis. A few shortcomings, however, have to be addressed before it can be used in a clinical setting. Though the circumferential strains are accurately estimated by CIV, radial strains (shown in Fig. 9) are underestimated. This could be due to (i) the thinness of the ventricular wall, owing to which the number of grid points in the radial direction is small in the region, and (ii) relatively smaller displacements in some segments, which leads to larger errors in the CIV estimates. The accuracy of the strain estimates also depend on the extent of smoothing done during the CIV processing and the treatment of data close to the domain boundaries; these processing protocols have to be suitably optimized for clinical applications. Interestingly, an underestimation of the radial strains have been reported for other methodologies too [23]. Also, the processing pipeline can be optimized to minimize the computational time. Currently, on a standard laptop (8 GB RAM, Processor: Intel Core i5-3230M CPU @ 2.6 GHz), HARP takes around 5–10 s to perform calculations over an entire cardiac cycle for a single slice on a single patient; in comparison, our technique takes around 45 s to perform the same calculations. We would like to point out that the software UVMAT is implemented on Matlab, and there is certainly scope for improvement in our computational times. In addition, the segmentation of the myocardium can be automated using algorithms similar to what is described in [28]. Furthermore, parallelization of the implementation of CIV is another avenue for reducing overall processing times. A clinical trial of patients with variable heart function would be required before routine clinical use.

## Conclusion

In this paper, we have demonstrated the ability of the CIV algorithm to estimate cardiac deformation from tMR images, and the resulting displacement and strain fields were found to be physiologically consistent. Our circumferential strain estimates are in good agreement with those of HARP in most regions of the heart, but the radial strains are underestimated. Our method fundamentally differs from HARP wherein the displacement fields are directly estimated from the phase image. Here we use the harmonic phase image to extract the grid into a grayscale image in successive time frames and then estimate the grid deformation using the CIV algorithm. It is noteworthy that we neither require a shape template nor a numerically complicated registration optimization algorithm.

With regards to our other specific objectives, we have

- extracted the grid from tMR images using Fourier techniques and used it as input to CIV algorithms. Our grid extraction algorithm was found to be robust by successful implementation on synthetic, phantom and patient images,
- identified that (*C*_*B*_,*S*_*B*_)=(25,55) pixels represent the optimum CIV algorithm parameters that result in less than 4% error for pixel displacements larger than 3 pixels on a 512 × 512 pixels image,
- shown that the estimated displacements of individual landmarks in patient images agree with expert raters to within 1 pixel on an average,
- demonstrated agreement with HARP on circumferential strain rates in most of the AHA model segments. The radial strain rates, however, were underestimated in comparison with HARP.

An analysis of the strain curves in arbitrarily defined segments of the myocardium would be relevant for clinical trials, particularly in quantifying the difference between healthy and diseased hearts. We have presented a 2D analysis of cardiac deformation even though the motion in 3D in nature. Despite the 2D nature of the analysis the method offers the flexibility to analyze multiple views (sagittal, coronal) thus enabling a pseudo-3D analysis. The extension of this method to fully 3D analysis will be the subject of future work.

## Additional file

Additional file 1 Appendix. Details of CIV algorithm and UVMAT software. (PDF 1720 kb)

## Abbreviations

CIV: Correlation image velocimetry
HARP: Harmonic phase analysis
LV: Left ventricle
PIV: Particle image velocimetry
STACOM: Statistical atlases and computational models of the heart: Imaging and modelling challenges
TMR: Tagged magnetic resonance
