# Supplementary material for: Estimation of myocardial deformation using correlation image velocimetry
Source: BMC Med Imaging. 2017 Apr 5;17:25. doi: 10.1186/s12880-017-0195-7 (PMC5382518; doi:10.1186/s12880-017-0195-7)
Supplement: Additional file 1 — Appendix. Details of CIV algorithm and UVMAT software. (PDF 1720 kb) [file 12880_2017_195_MOESM1_ESM.pdf]

Appendix

In this appendix, we describe the *UVMAT* processing pipeline (summarised in figure 2) starting from the image pair input to the estimation of a dense displacement field at a given time t_0. The pre processed image pair, corresponding to times t_0 and $t_0 + \Delta t$, are first loaded into the software. Δt is chosen based on the constraints discussed in the **Discussion** section. A typical screenshot of the PIV menu of the *UVMAT* GUI where the user specifies the various inputs is shown in figure A1. Using the option 'Mask', an approximate segmentation mask that can be used throughout the cardiac cycle is created manually. While several input image formats are supported, the outputs are in the standard netcdf format. It is also possible to save the output data in other formats from the Matlab command prompt.

As mentioned earlier, the CIV processing is implemented as a hierarchical process, with the first step performing a coarse estimation of the displacement field. Each step involves performing cross-correlation (denoted by civ1 & civ2 in figure A1), followed by outlier elimination (fix1 & fix2) and spatial smoothing (patch1 & patch2).

- civ1: the first (coarse) image correlation. In this step, the cross-correlation between Image 1 (with intensity distribution I_a) and Image 2 (with intensity distribution I_b) within a correlation box for a pixel displacement of (i, j) is computed as [1, 2]:

$$c(i,j) = \frac{\Sigma_{k=1}^{C_B}\Sigma_{l=1}^{C_B}(I_a(k,l) - \bar{I_a})(I_b(k+i,l+j) - \bar{I_b})}{[\Sigma_{k=1}^{C_B}\Sigma_{l=1}^{C_B}(I_a(k,l) - \bar{I_a})^2\Sigma_{k=1}^{C_B}\Sigma_{l=1}^{C_B}(I_b(k+i,l+j) - \bar{I_b})^2]^{1/2}}, \tag{1}$$

  where $\bar{I_a}$ and $\bar{I_b}$ are the spatial means of I_a and I_b within the correlation box. The range of displacements (i, j) for which $c(i, j)$ is computed is specified by S_B. The first estimate of the single displacement representative of the entire correlation box is given by the (i, j) values for which c attains a maximum. Sub-pixel accuracy is achieved for the estimation of the location of the maximum via thin-plate 2D interpolation of $c(i, j)$.

- fix1: detection of 'false' vectors in civ1 displacement field. The cross-correlation fields from two adjacent correlation boxes are cross-correlated to identify false maxima. Since neighboring regions are expected to have nearby maxima locations, this step will cancel out the false maxima arising due to noise. Manual removal of spurious velocity vectors is also possible but not recommended for large datasets.

- patch1: interpolation using thin plate spline and filtering on a regular grid, providing access to spatial derivatives of the displacement. The civ1 step assigns a displacement vector to the center pixel of every correlation box, thus providing a displacement gradient field. The intensity values of the image patch inside the correlation box is fit to a thin-plate spline, following which the gradient of the displacement field is used to deform/warp the image patch [3].

- civ2: the second pass of the hierarchical image correlation process using the result of civ1 as a first approximation to output an improved estimate of the displacement field. The deformed correlation box is used to identify the cross-correlation maxima similar to civ1 but in this case the search area is restricted to a couple of pixels around the maxima location determined in civ1.

- fix2 and patch2: similar to fix1 and patch1, but applied to the civ2 results.

The process outlined above can be repeated to obtain the desired level of accuracy in the location of the maxima and consequent displacement field estimates. The parameters used in the aforementioned operations are given in table A1. The displacement field is then projected onto the desired grid using 'merge_proj' option in the 'field series' menu of *UVMAT* to get the final interpolated displacements at every pixel. The parameters used in the same are given in table A2.

In summary, *UVMAT* is a free open source software written in Matlab, and its interface offers an easy to use tool for estimating cardiac deformations from tMR images. Displacement fields can be estimated from tMR images of the heart with minimal pre-processing, and finally be exported in any desired format. Eulerian strains can then be numerically estimated using finite difference approximation from the displacement fields. Instances of successful implementation of the software in fluid flow experiments can be found in [4].

References

[1] Fincham, A.M., Spedding, G.R.: Low cost, high resolution dpiv for measurement of turbulent fluid flow. Experiments in Fluids **23**, 449–462 (1997)

[2] Sommeria, J.: UVMAT. http://servforge.legi.grenoble-inp.fr/projects/soft-uvmat/

[3] Fincham, A., Delerce, G.: Advanced optimization of correlation imaging velocimetry algorithms. Experiments in Fluids **29**(1), 013–022 (2000). doi:10.1007/s003480070003

[4] Sommeria, J.: Correlation Imaging Velocimetry at the Coriolis Facility. http://servforge.legi.grenoble-inp.fr/projects/soft-uvmat/attachment/wiki/WikiStart/CIV_doc_lim.pdf. Accessed: 18-November-2015 (2003)

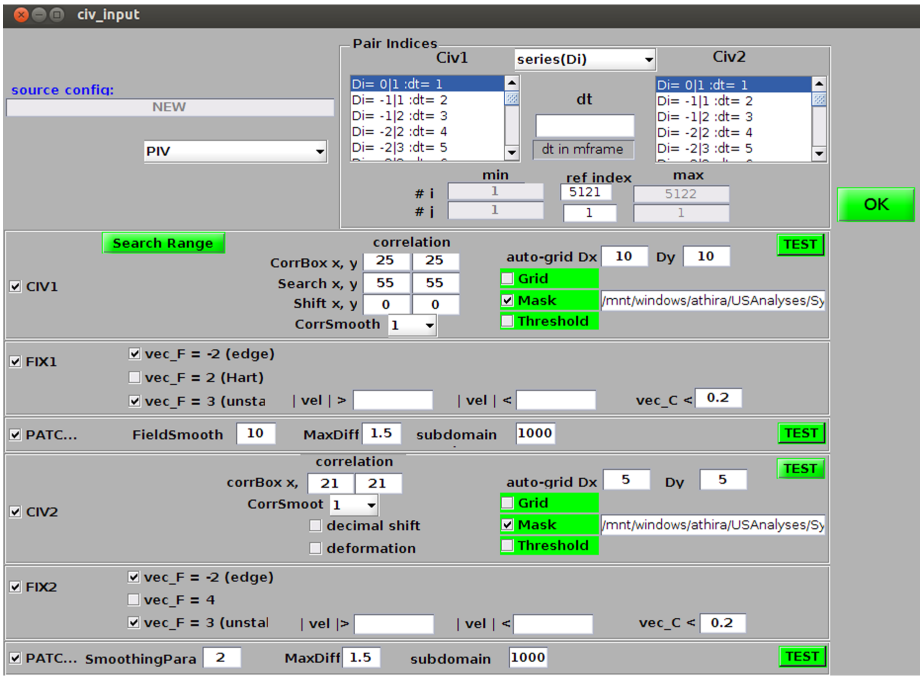

Figure A1: A typical screenshot from the *UVMAT* software.

Parameter	Value
CorrBoxSize (C_B)	25 x 25
SearchBoxSize (S_B):	55 x 55
Auto Grid Dx:	10 x 10
Mask	Select mask image

Table A1: Parameters and their corresponding values for CIV

Parameter	Explanation	Value
Type	Classify the object on which the field is projected.	Plane
ProjMode:	Specifies the method of projection of coordinates and field	'interp_tps'
RangeX	Bounds defining a range of projection along X coordinate with respect to the object.	0 - 512
RangeY	Bounds defining a range of projection along Y coordinate with respect to the object.	0 - 512
Dx	Mesh along X coordinate defining a grid for interpolation.	1
Dy	Mesh along Y coordinate defining a grid for interpolation.	1

Table A2: Parameters and their corresponding values for interpolation for 'merge_proj'
